# Progression of diabetes is associated with changes in the ileal transcriptome and ileal‐colon morphology in the UC Davis Type 2 Diabetes Mellitus rat

**DOI:** 10.14814/phy2.15102

**Published:** 2021-11-21

**Authors:** Brian D. Piccolo, James L. Graham, Ping Kang, Christopher E. Randolph, Kartik Shankar, Laxmi Yeruva, Renee Fox, Michael S. Robeson, Becky Moody, Tanya LeRoith, Kimber L. Stanhope, Sean H. Adams, Peter J. Havel

**Affiliations:** ^1^ USDA‐ARS Arkansas Children's Nutrition Center Little Rock Arkansas USA; ^2^ Department of Pediatrics University of Arkansas for Medical Sciences Little Rock Arkansas USA; ^3^ Department of Molecular Biosciences School of Veterinary Medicine University of California Davis Davis California USA; ^4^ Department of Nutrition University of California Davis Davis California USA; ^5^ Center for Translational Pediatric Research Arkansas Children's Research Institute Little Rock Arkansas USA; ^6^ Department of Pediatrics Section of Nutrition University of Colorado School of Medicine Anschutz Medical Campus Aurora Colorado USA; ^7^ Arkansas Children's Research Institute Little Rock Arkansas USA; ^8^ Department of Biomedical Informatics University of Arkansas for Medical Sciences Little Rock Arkansas USA; ^9^ Department of Biomedical Science and Pathobiology Virginia Polytechnic Institute and State University Blacksburg Virginia USA; ^10^ Department of Surgery University of California Davis School of Medicine Sacramento California USA; ^11^ Center for Alimentary and Metabolic Science University of California Davis School of Medicine Sacramento California USA

**Keywords:** crypt, diabetes, microbiome, RNA‐seq, UCD‐T2DM rat, villi

## Abstract

Deterioration in glucose homeostasis has been associated with intestinal dysbiosis, but it is not known how metabolic dysregulation alters the gastrointestinal environment. We investigated how the progression of diabetes alters ileal and colonic epithelial mucosal structure, microbial abundance, and transcript expression in the University of California Davis Type 2 Diabetes Mellitus (UCD‐T2DM) rat model. Male UCD‐T2DM rats (age ~170 days) were included if <1‐month (*n* = 6, D1M) or 3‐month (*n* = 6, D3M) post‐onset of diabetes. Younger nondiabetic UCD‐T2DM rats were included as a nondiabetic comparison (*n* = 6, ND, age ~70 days). Ileum villi height/crypt depths and colon crypt depths were assessed by histology. Microbial abundance of colon content was measured with 16S rRNA sequencing. Ileum and colon transcriptional abundances were analyzed using RNA sequencing. Ileum villi height and crypt depth were greater in D3M rats compared to ND. Colon crypt depth was greatest in D3M rats compared to both ND and D1M rats. Colon abundances of *Akkermansia* and Muribaculaceae were lower in D3M rats relative to D1M, while *Oscillospirales*, *Phascolarctobacterium*, and an unidentified genus of Lachnospiraceae were higher. Only two transcripts were altered by diabetes advancement within the colon; however, 2039 ileal transcripts were altered. Only colonic abundances of *Sptlc3*, *Enpp7*, *Slc7a15*, and *Kctd14* had more than twofold changes between D1M and D3M rats. The advancement of diabetes in the UCD‐T2DM rat results in a trophic effect on the mucosal epithelia and was associated with regulation of gastrointestinal tract RNA expression, which appears more pronounced in the ileum relative to the colon.

## Graphical abstract

The advancement of diabetes in the UC Davis Type 2 Diabetes Mellitus (UCD‐T2DM) rat model results in a trophic effect on the mucosal epithelia. Diabetes was also associated with regulation of gastrointestinal tract RNA expression, which appears more pronounced in the ileum relative to the colon. The UCD‐T2DM rat spontaneously develops diabetes while consuming standard chow, thus, these changes may be diabetes specific.

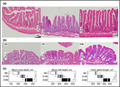

## 1 INTRODUCTION

Type 2 Diabetes Mellitus (T2DM) is intrinsically linked to both genetic predisposition and to lifestyle factors that promote fat deposition, and it is thought that T2DM can be prevented or treated with diet and physical activity changes (Kolb & Martin, 2017). Still, understanding the etiology and symptoms of T2DM is key to developing strategies for early diagnosis, management, and mitigation of complications.

Considerable attention has been focused on the co‐morbidities of T2DM including cardiovascular disease, obesity, hyperlipidemia, and chronic kidney disease, since these contribute to the greatest disability and mortality associated with T2DM (Iglay et al., 2016); however, there are several other complications of T2DM that are not fully understood. For example, T2DM is associated with several gastrointestinal disorders, including gastroparesis and enteral neuropathy (Boaz et al., 2011; Quan et al., 2008), both of which can manifest clinically as diarrhea, constipation, and/or fecal incontinence (Krishnan et al., 2013). These complications can result in a lower quality of life and in some cases, social fear or embarrassment (Woodhouse et al., 2017). While the mechanisms underlying the clinical manifestations have yet to be clearly elucidated, the pathogenesis of gastrointestinal comorbidities associated with diabetes are likely a result of oxidative stress, neuropathy, and changes in the biomechanical properties of the intestine (Du et al., 2018). More recently, T2DM has also been associated with dysbiosis of the gut microbiome (Karlsson et al., 2013; Qin et al., 2012) and others have speculated that the gut dysbiosis associated with obesity may contribute to insulin resistance (Cani et al., 2007, 2008). Despite evidence for an association between T2DM and alimentary tract dysfunction, availability of literature describing alterations in the morphology and phenotype of the gastrointestinal tract during the progression of T2DM is quite limited. Furthermore, it can be difficult to deconvolute the direct effects of T2DM itself and the poor quality dietary patterns that can often accompany the development of the disease.

Several reports have noted histomorphometric remodeling in both small and large intestines from both genetic and experimentally induced animal models of diabetes (Bhor et al., 2004). Results have generally been consistent, with increased mucosal thickness, intestinal wall stiffening, and expansion of intestinal villi (Adachi et al., 2003; Chen et al., 2012). Other modifications of gastrointestinal cells have been observed, including an increase in inflammation (Ding et al., 2010) and gut permeability (Cani et al., 2008). Many of these studies have been done in chemically induced diabetic animal models, which does not account for the polygenic origin of type 2 diabetes. We have recently observed changes in the cecal microbiome and metabolome in the UC Davis Type 2 Diabetes Mellitus (UCD‐T2DM) rat model, coincident with diabetes progression (Piccolo et al., 2018). The UCD‐T2DM rat spontaneously develops overt diabetes while consuming a standard chow diet and possesses a number of clinical features that more closely model human T2DM than other rodent models of the disease. For example, UCD‐T2DM rats have intact leptin signaling, develop diabetes in both sexes, and females maintain their fertility (Cummings et al., 2008). As no dietary intervention is required to initiate diabetes in this model, it was hypothesized that the observed changes in the cecal microbiome are likely driven by diabetes‐related alterations to the gastrointestinal environment (Piccolo et al., 2018). Hansen et al. previously described mucosal hypertrophy in jejunal and ileal segments after ileal–jejunal transposition surgery in 3.5 month old UCD‐T2DM rats (Hansen et al., 2014); however, histomorphometric alterations related to the advancement of diabetes has not been documented in this model. Thus, our aim in this study was to determine how advanced diabetes contributes to dysbiosis of the gastrointestinal tract of UCD‐T2DM rats.

## 2 METHODS

### 2.1 Rats

Male UCD‐T2DM rats ~170‐day old that were either recently diagnosed with diabetes (D1M; *n* = 6; nonfasting blood glucose > 300 mg/dl for two consecutive weeks) or had untreated diabetes for 3 months (D3M, *n* = 6) were included in this study. The UCD‐T2DM rat colony has been maintained at the animal facility in the Department of Nutrition at UC Davis for >15 years and are maintained on a 14:10 h light–dark cycle with ad libitum access to standard chow (2018 Telkad Global; Harlan Laboratories). The UCD‐T2DM rat model has no genetically similar control, therefore, UCD‐T2DM rats without diabetes were also sampled as a nondiabetic controls (*n* = 6; ~70 days old) for comparison with the diabetic animals. Rats were euthanized after a 13 h fast by exsanguination following a 200 mg/kg intraperitoneal injection of pentobarbital sodium. All animal protocols were approved by the Institutional Animal Care and Use Committee of the University of California Davis (protocol 20092).

### 2.2 Intestinal samples

Immediately following euthanasia, the entire gastrointestinal tract from the duodenum to the rectum was dissected, removed, weighed, and then placed on a sterile tray on ice. The length of the gastrointestinal tract was measured and then the distal ileum and proximal colon were collected. Two‐centimeter sections of ileum and colon samples were placed in 10% formalin for histomorphometric analysis. An additional 2 cm section of ileum and colon tissue were placed in a conical tube and immediately flash frozen in liquid nitrogen. Frozen tissues were moved to −70°C for storage until analysis.

### 2.3 Histology

Formalin‐fixed tissues were cut, paraffin‐embedded, and 6‐μm thick sections were processed for staining with hematoxylin and eosin (H and E). Transversely oriented tissue sections were examined at 40× magnification using a Nikon Eclipse Ti2 microscope. Images of whole tissue sections were captured at 40× using Nikon NIS‐Elements software (version 5.21.02). ImageScope (version 12.1.0.5029; Aperio Technologies) software was used to measure the ileal villus height, crypt density, and colon granular muscularis.

### 2.4 Real‐time polymerase chain reaction

RNA was extracted from whole ileum and colon tissues using TRIzol reagent and total RNA was isolated using RNAeasy mini columns (Qiagen) following the manufacturer's instructions. Total RNA (1 μg) was reverse transcribed using the iScript cDNA synthesis kit (Bio‐Rad). Real‐time polymerase chain reaction (rtPCR) was performed using an ABI Prism 7500 Fast instrument. Relative amounts of mRNA were quantified using the delta delta cycle threshold (ddCT) quantification method (Livak & Schmittgen, 2001) and normalized to the expression of Ribosomal protein L13A (Rpl13a) mRNA (Jonge et al., 2007). Gene‐specific primers were designed with Primer Express Software (Table 1).

**TABLE 1:** Primer sequences used for real time PCR

Gene	Forward	Reverse
Cldn1	CTGTGGTAGAACAAAAGCAAGCA	AAAGGCTTCCCTCCTG TACTCA
Cldn3	TCTGCTTGCTAGGCTGGAAGA	TTGTCCATTCGACTTGGACAGT
Cldn4	CCTTTCCCATACGGTCTTGCT	TGGACAAGGGTAGGGAATTCAG
Cldn5	GGACTTGACCGACCTTTTCTTCT	GGAACTGTTAGCGGCAGTTTG
Cldn8	ATGACTCCCTGCTGGCTCTTAG	GAGGATGGCTGTCATGAAAGC
Cldn12	TAGCCTGGACTCTGGGAATTCA	TCTCTCTCCTATCTCGCCCATT
Ocln	GACATCAGCCATGTCTGTGAGG	CGCCATACATGTCATTGCTTG
Il1a	GGCCATAGCCCATGATTTAGAA	CCTGCTTGACGATCCTTATCAAT
Pai1	TTCATGCCCCACTTCTTCAAG	CTCTCCACCCAGTCGTTGATG
Rpl13a	GGATCCCTCCACCCTATGACA	ACGCCCCAGGTAAGCAAACT

### 2.5 RNA‐seq analysis

Sequence ready cDNA libraries were prepared using total RNA from ileum and colon tissue using the Illumina TruSeq stranded mRNA Sample Prep kit, following the manufacturer's protocol. RNA samples were randomized and re‐assigned labels prior to library prep. Poly‐A containing mRNA was selected using oligo‐dt attached magnetic beads and then converted to first strand cDNA using reverse transcription and random primers. A second strand was then synthesized to produce double stranded cDNA, followed by fragmenting and end repair. A single ‘A’ nucleotide was added to the 3′ ends of the cDNA allowing the ligation of unique index adapters. The cDNA libraries were PCR enriched and then checked for fragment size and concentration using an Agilent Bioanalyzer and a Thermofisher Qubit fluorometer. Libraries were normalized by molarity and samples were pooled at 4 nM. The pooled libraries were denatured and diluted to a concentration of 1.8 pM prior to sequencing. RNA sequencing was performed using an Illumina NextSeq 500 series sequencer. Generated raw data were uploaded in real time to the Illumina BaseSpace Squencing Hub for binar base call (BCL) to FastQ file conversion. Data analysis was performed via alignment of high‐quality reads using spliced transcripts alignment to a reference (STAR). Resulting read alignments for each sample were imported in Seqmonk for gene level quantification as counts mapping to annotated genes. RNA‐seq data were deposited to the Gene Expression Ominbus repository (GEO181275). Gene counts were imported into R for differential expression analysis.

### 2.6 16S rRNA amplicon sequencing

Bacterial DNA was isolated from ileum and colon contents (0.1–0.3 g) using the DNeasy PowerSoil HTP 96 Kit (Qiagen) following the manufacturer's instructions. Following the methods of Kozich et al., extracted DNA (50 ng) was used for amplification of the variable region 4 (V4) of the 16S rRNA gene using 515F/806R primers via PCR. Pooled amplicons were then paired‐end sequenced (2 × 250 bp) using an Illumina MiSeq with ~30% of PhiX DNA. Barcodes were extracted from the I1 and I2 fastq files using the extract_barcodes.py function in QIIME1 (v1.9.1) and then data were imported and demultiplexed in QIIME2 (Bolyen et al., 2019). Pseudo and pooled arguments were selected for pooling‐ and chimera‐methods. All other DADA2 settings were set to default. Taxonomic assignment was assessed using a RESCRIPt prepared Naïve Bayes classifier for the V4 hyper variable regions, that is, the silva‐138‐99‐515‐806‐nb‐classifier (Bokulich et al., 2018; Robeson et al., 2020). Phylogenetic trees were constructed using the RAxML rapid bootstrap procedure (Stamatakis et al., 2008). Microbial sequencing data have been deposited at the Sequencing Read Archive, accession number PRJNA743666. Data are reported as amplicon sequence variant (ASV) counts. Count table, taxonomy table, and phylogenetic tree were all created in QIIME2 and then exported to the R Statistical Language for further analysis.

### 2.7 Statistical analysis

All statistical analyses, figures, and tables were conducted in the R Statistical Language (version >4.0.3). Statistical significance for non‐omics data was set at *α* < 0.05. Age parameters, body weight, blood glucose, and colon length measurements had non‐normal distribution based on Shapiro–Wilk tests, and were assessed for group differences using a Kruskal–Wallis test followed by Dunn's test of multiple comparisons using rank sums. Histological data had a normal distribution and were assessed with ANOVA followed by Tukey's honest significance difference (HSD) test. Linear or polynomial trends in histological data were assessed with specific contrasts. Beta‐diversity was assessed with Bray–Curtis Dissimilarities and Unweighted Unifrac Distances, and visualized with Principal Co‐ordinate Analysis (PCoA). Group differences in beta‐diversity were determined with permutational multivariate ANOVA (PERMANOVA) with 999 permutations. Pairwise assessment of individual taxa was conducted at the genus level using negative binomial generalized linear models (Love et al., 2014). Differential analysis of RNA‐seq data was performed using the edgeR pipeline (McCarthy et al., 2012; Robinson et al., 2010). *p*‐values obtained from sequencing data analyses were corrected for multiple comparisons using the Benjamini and Hochberg (1995) method and considered significant at false discovery rate (FDR) < 0.05. Pathway analysis on significant transcripts was conducted using the DAVID Bioinformatics Resource 6.8 (Huang et al., 2009, 2009). Correlations between ASVs and transcripts were assessed with Spearman's correlations.

## 3 RESULTS

Characteristics of the rats included in this study are presented in Table 2. The age of D1M and D3M rats at sacrifice differed by ~3 days, but the difference in total days diagnosed with diabetes between D3M and D1M rats was 79 days. ND rats were statistically younger than both D1M and D3M rats, as indicated in the methods. Total body weight was greater in D1M and D3M rats relative to ND rats; however, D3M rats had lower total body weight compared with D1M. As previously noted, the latter observation indicates weight loss associated with more advanced diabetes in this model (Cummings et al., 2008; Piccolo et al., 2016). Fasting blood glucose was 2× higher in D3M rats compared with D1M rats. While median glucose was higher in D1M rats compared with ND rats, this did not reach statistically significance (*p* = 0.065). Colon length was greater in diabetic rats, relative to ND rats. When adjusting for total body weight, D1M rats had smaller colon lengths relative to ND and D3M rats.

**TABLE 2:** Characteristics of male UC Davis Type 2 Diabetes Mellitus rats^1^

Parameters	ND	D1M	D3M
Age, days	70 (0)^a^	169 (1.6)^b^	172.3 (0.5)^c^
Age of diabetes onset, days		146.7 (1.6)^b^	71 (8.9)^a^
Days with diabetes		22.3 (0.5)^a^	101.3 (8.9)^b^
Body weight, g	414.3 (16.3)^a^	634.2 (25.9)^c^	517.5 (38.5)^b^
Fasting glucose, mg/dl	94.7 (5.9)^a^	123.3 (34.2)^a^	266.2 (67.1)^b^
Colon length, cm	23.0 (1.2)^a^	26.3 (1.3)^b^	27.6 (1.7)^b^
Colon length^2^	0.06 (0)^b^	0.04 (0)^a^	0.05 (0.01)^b^

^1^ UCD‐T2DM rats were non‐diabetic (ND, *n* = 6), had recent diabetes (2‐week post‐onset of diabetes; D1M, *n* = 6), or 3‐month post‐onset of diabetes (D3M, *n* = 6). Means without a common letter differ with the use of Dunn's test of multiple comparisons using rank sums.

^2^ Adjusted for total body weight.

### 3.1 Ileum and colon mucosal structure

**FIGURE 1:**
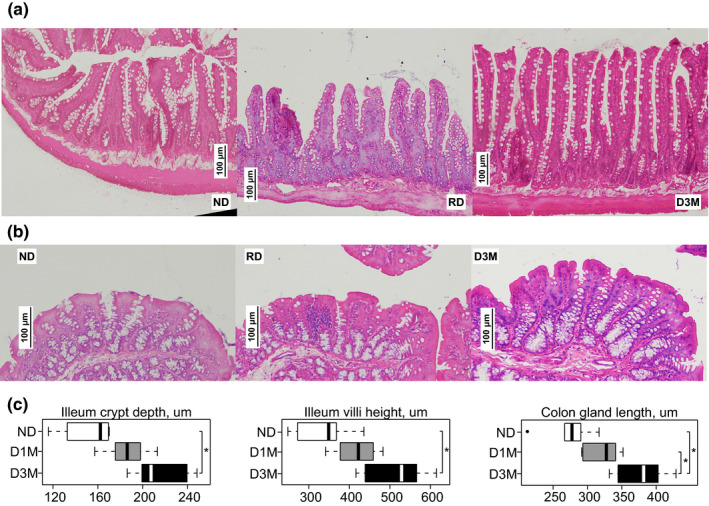
Histomorphometric analysis of (a) ileum and (b) colon epithelia from UC Davis Type 2 Diabetes Mellitus (UCD‐T2DM) rats. Data are median and IQR (c) of UCD‐T2DM rats that are either non‐diabetic (ND, *n* = 6), recent onset of diabetes (D1M, *n* = 6), and 3‐month post‐onset of diabetes (D3M, *n* = 6). Twenty measurements were assessed per slide and then averaged for final estimate. Ileum crypt depth and villi height are indicated in micrometers on x‐axis of boxplots. Colon crypt depth is indicated in micrometers on *x*‐axis of boxplots. Group differences assessed by ANOVA followed by Tukey's honestly significant difference (HSD) test. * indicates statistical significance, *p* < 0.05

Ileal crypt depth was greater in diabetic rats, compared with ND rats (Figure 1A). While ileal crypt depths in D1M and D3M rats were not statistically different from each other, a linear trend following the advancement of diabetes duration was observed (*p* < 0.01). The only statistically significant difference in ileal villi height was observed between ND and D3M (Figure 1A). However, a significant linear trend was observed between the groups with increasing villi height tracking advancement of diabetes duration (*p* < 0.01). Colonic crypt depth was greatest in D3M rats relative to ND and D1M rats (Figure 1B). Similar to observations in the ileum, colonic crypt depth increased linearly as the duration of diabetes advanced (*p* < 0.01).

### 3.2 Microbiota

**FIGURE 2:**
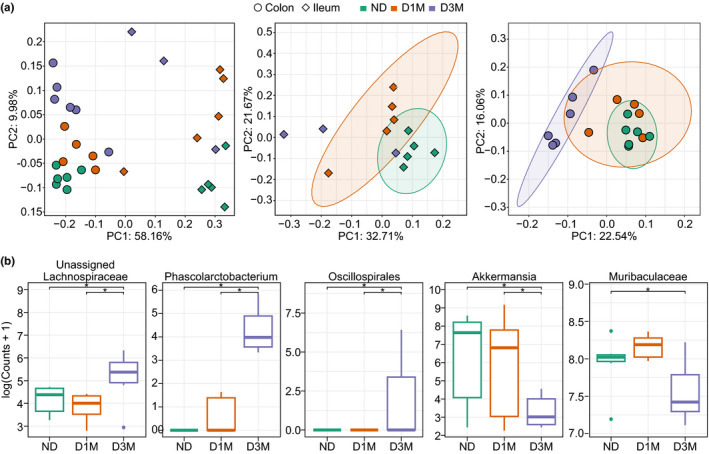
Bacterial community analysis of ileum and colon contents from UC Davis Type 2 Diabetes Mellitus (UCD‐T2DM) rats that were non‐diabetic (ND), 2‐week post‐onset of diabetes (D1M), or 3‐month post‐onset of diabetes (D3M). Beta‐diversity was assessed with Bray–Curtis Dissimilarities and visualized with Principal Co‐ordinate Analysis (PCoA). Statistical significance was determined with permutational multivariate ANOVA, *p* < 0.05. PCoA of (a) all samples, ileum samples, and colon samples. (b) Differential expressed genus taxonomy identified in colon contents. Data are log(read counts + 1). Differential analysis was conducted using the DESeq2 R Bioconductor package. Statistical significance determined with Benjamini and Hochberg corrected *p*‐value at *p*_adjusted_ < 0.05. Ileum sample size: ND = 5, D1M = 5, D3M = 3; Colon sample size: ND = 6, D1M = 5, D3M = 5. Ileum D3M samples included PCoA for visual purposes, but were not included in statistical tests

Median sample depth of raw sequencing reads was lower in ileal samples relative to colon, irrespective of diabetes status (ileum: median = 6464[IQR = 5206]; colon: 12070[3457]). Two ileum samples and one colon sample failed to sequence (sample depths < 100 counts) and an additional three ileal samples had low depth (<5000 counts); these samples were removed from the analysis. Of the six ileum samples that were removed, three of them were D3M rats. Therefore, ileal samples from D3M rats were kept in non‐supervised ordinations, but were not included in subsequent statistical analyses. Bray–Curtis Dissimilarities clearly separated ileum and colon samples along PC1 (58.2% explained variance) of the PCoA (Figure 2A). When assessing diabetes status within intestinal regions, ND and D1M were significantly different in the ileum (Figure 2A; *p* < 0.01), while D3M separated from both ND and D1M groups in the colon. In the ileum, the *Romboutsia* genus was greater in D1M versus ND rats while the RF39 genus was lower in D1M (Table 3). Colonic genera that differed between ND and either D1M and D3M groups are presented in Table 3. Only five genera differed in the colon between D1M and D3M groups (Figure 2B): *Akkermansia* and Muribaculaceae were lower in D3M rats compared to D1M rats, while Oscillospirales, *Phascolarctobacterium*, and an unidentified genus in the Lachnospiraceae family were greater in D3M rats.

**TABLE 3:** Differentially abundant amplicon sequence variant (ASV) in ileum and colon contents from male UC Davis Type 2 Diabetes Mellitus (UCD‐T2DM) rats^a^

Phylum	Family	Genus	Base mean^b^	log2 fold difference^c^	*p*_adj_ ^d^	Comparison^e^
Ileum						
Firmicutes	*RF39*	*RF39*	18.5	6.4	0.02	D1M versus ND
Firmicutes	Peptostreptococcaceae	*Romboutsia*	1836.4	−3.6	<0.01	D1M versus ND
Colon						
Firmicutes	Lachnospiraceae		116.9	1.9	0.01	D3M versus ND
Verrucomicrobiota	Akkermansiaceae	*Akkermansia*	1411.1	−5.4	<0.01	D3M versus ND
Firmicutes	Acholeplasmataceae	*Anaeroplasma*	5.0	−5.9	0.03	D3M versus ND
Actinobacteriota	Bifidobacteriaceae	*Bifidobacterium*	697.3	3.3	<0.01	D3M versus ND
Firmicutes	Ruminococcaceae	*CAG‐352*	118.9	25.5	<0.01	D3M versus ND
Firmicutes	*Clostridia_vadinBB60_group*	*Clostridia_vadinBB60_group*	31.2	−5.7	0.01	D3M versus ND
Actinobacteriota	Corynebacteriaceae	*Corynebacterium*	21.1	5.8	0.01	D3M versus ND
Firmicutes	Erysipelotrichaceae	*Dubosiella*	346.0	2.2	0.03	D3M versus ND
Proteobacteria	Enterobacteriaceae	*Escherichia‐Shigella*	59.3	4.8	<0.01	D3M versus ND
Campilobacterota	Helicobacteraceae	*Helicobacter*	10.2	−3.9	0.03	D3M versus ND
Firmicutes	Oscillospiraceae	*Intestinimonas*	61.5	−4.2	0.02	D3M versus ND
Firmicutes	Lachnospiraceae	*Lachnospiraceae_FCS020_group*	3.2	−5.0	0.04	D3M versus ND
Firmicutes	Oscillospiraceae	*NK4A214_group*	42.7	1.7	0.02	D3M versus ND
Firmicutes	Oscillospiraceae	*Oscillibacter*	118.7	2.6	0.01	D3M versus ND
Firmicutes	Oscillospirales	*Oscillospirales*	48.9	24.7	<0.01	D3M versus ND
Bacteroidota	Tannerellaceae	*Parabacteroides*	13.8	−2.4	0.01	D3M versus ND
Firmicutes	Acidaminococcaceae	*Phascolarctobacterium*	39.6	9.4	<0.01	D3M versus ND
Firmicutes	Ruminococcaceae	*Ruminococcaceae*	2.6	−3.6	0.02	D3M versus ND
Firmicutes	Ruminococcaceae	*Ruminococcus*	115.7	−1.5	0.03	D3M versus ND
Firmicutes	Erysipelotrichaceae	*Turicibacter*	38.4	2.1	0.01	D3M versus ND
Firmicutes	Ruminococcaceae	*uncultured*	12.4	−1.2	0.04	D3M versus ND
Bacteroidota	Barnesiellaceae	*Barnesiella*	7.9	−3.3	0.04	D1M versus ND
Actinobacteriota	Bifidobacteriaceae	*Bifidobacterium*	697.3	4.2	<0.01	D1M versus ND
Firmicutes	Ruminococcaceae	*CAG‐352*	118.9	23.2	<0.01	D1M versus ND
Firmicutes	Clostridia_UCG‐014	*Clostridia_UCG‐014*	109.2	3.1	0.03	D1M versus ND
Firmicutes	Erysipelotrichaceae	*Dubosiella*	346.0	2.7	0.03	D1M versus ND
Firmicutes	RF39	*RF39*	61.0	3.4	0.01	D1M versus ND
Firmicutes	Ruminococcaceae	*Ruminococcus*	115.7	−2.7	<0.01	D1M versus ND

^a^ UCD‐T2DM rats were non‐diabetic (ND, *n* = 6), had recent diabetes (2‐week post‐onset of diabetes; D1M, *n* = 6), or 3‐month post‐onset of diabetes (D3M, *n* = 6). Analysis conducted with the DESeq2 R package.

^b^ Normalized counts of all samples adjusting for sequencing depth.

^c^ Effect size estimate.

^d^ Bonferroni and Hochberg corrected *p*‐value.

^e^ Comparison for log twofold difference. Second listed group is the base comparison.

### 3.3 Colonic gap junctions

**FIGURE 3:**
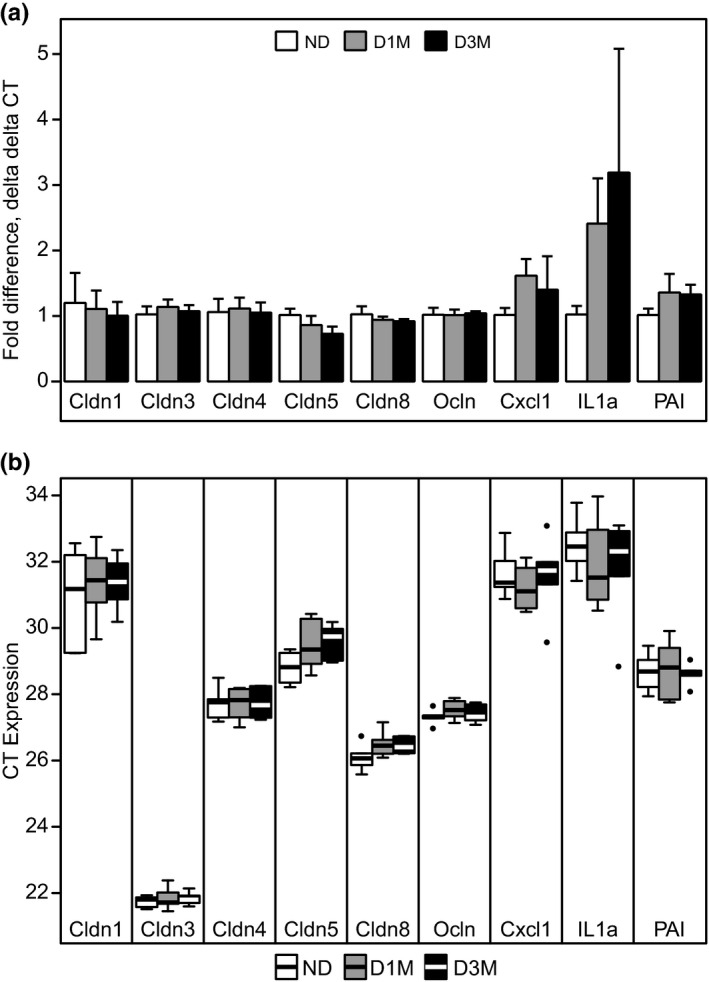
Colon gene expression levels of tight junction and markers of inflammation from UC Davis Type 2 Diabetes Mellitus (UCD‐T2DM) rats that were non‐diabetic (ND, *n* = 6), had recent diabetes (2‐week post‐onset of diabetes; D1M, *n* = 6), or 3‐month post‐onset of diabetes (D3M, *n* = 6). (a) Data are mean and SEM of fold differences using the delta delta CT method. ND rats were considered the control group to calculate delta delta CT. (b) Data are mean and standard error of the mean (SEM) of raw cycle threshold (CT) values. Kruskal–Wallis test was used to assess group differences at *p* < 0.05. No group differences were observed for any gene

We first focused our attention to genes associated with gut permeability in the colon since it has been reported that obesity is associated with increased gut permeability and hyperglycemia drives intestinal barrier dysfunction (Cani et al., 2008; Thaiss et al., 2018). However, we did not identify any differences between diabetic groups in the colonic expression of claudins or occludin using rtPCR analysis, nor did we identify differences in a limited set of inflammatory markers (Figure 3A). While there appears to be a large increasing trend in the fold differences of D1M and D3M IL11a levels compared to ND levels, the barplot does not take into account a single outlier in the D3M data (Figure 3B). Based on raw CT values, Cldn3 was the highest expressed claudin gene in the colon, followed by Cldn8, Cldn4, Cldn5, and Cldn1 (Figure 3B).

### 3.4 Global transcriptome analysis

**FIGURE 4:**
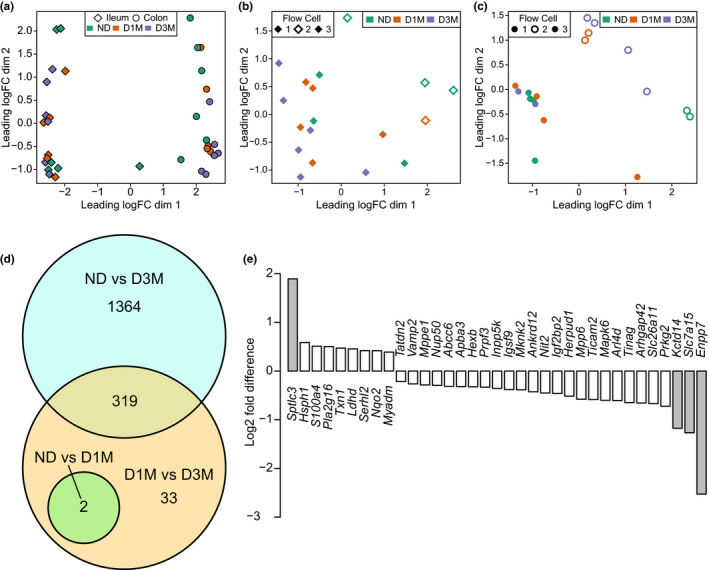
RNA sequencing analysis of ileal and colonic tissues from UC Davis Type 2 Diabetes Mellitus (UCD‐T2DM) rats that were non‐diabetic (ND, *n* = 6), had recent diabetes (2‐week post‐ onset of diabetes; D1M, *n* = 6), or 3‐month post‐onset of diabetes (D3M, *n* = 6). Global transcriptional abundances visualized with multidimensional scaling (MDS) with (a) all samples, (b) ileum samples, and (c) colon samples. Points indicate ileum (diamond) and colon (circles) tissues, colors discriminate rat group (ND: green; D1M: orange; D3M: purple). Differential analysis of RNA transcripts conducted using the edge2 R Bioconductor package. (D) Intersection of pairwise comparisons of ileum transcripts. Statistical significance determined with Benjamini and Hochberg corrected *p*‐value at *p*_adjusted_ < 0.05. (e) RNA transcripts that statistically differ between D1M and D3M rats, but do not intersect with other comparisons. Gray shaded bars indicate RNA transcripts with absolute log2 fold differences > 1

As there was no evidence of colonic gap junction dysfunction in the gene expression analysis, we then utilized RNAseq to capture a broader range of transcripts that are affected by the advancement of diabetes duration in this model. As expected, RNA expression data clearly separated ileal from colonic tissue (Figure 4A). However, when visualizing within intestinal region, separation between the diabetes groups was not readily apparent by PCA (Figure 4A). Furthermore, evidence of a batch effect was observed in both ileum and colon (Figure 4B,C), thus we performed pairwise comparisons to detect differences between all groups while controlling for batch effects between runs.

**FIGURE 5:**
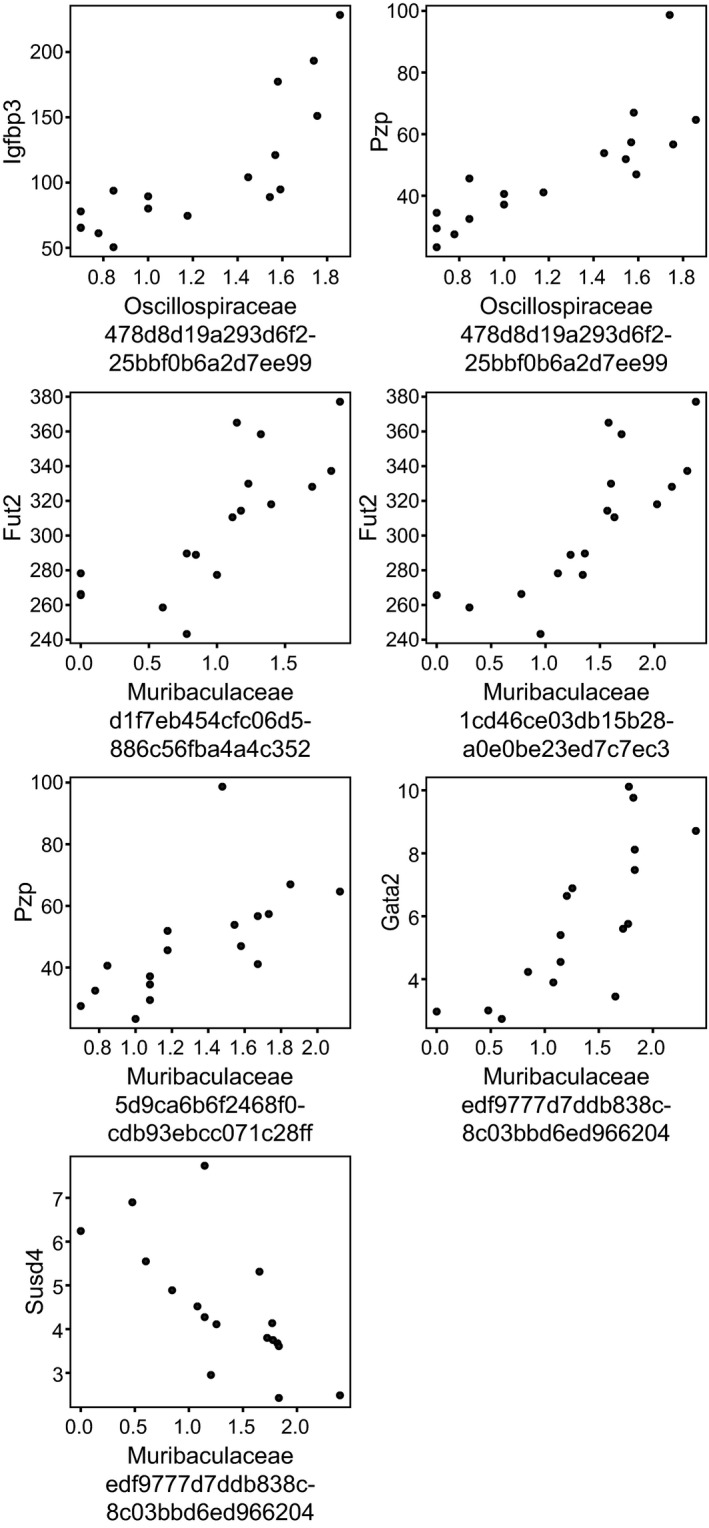
Correlation analysis of colonic content taxonomy and colon transcripts from UC Davis Type 2 Diabetes Mellitus (UCD‐T2DM) rats that were non‐diabetic (ND, *n* = 6), had recent diabetes (2‐week post‐onset of diabetes; D1M, *n* = 5), or 3‐month post‐onset of diabetes (D3M, *n* = 5). Microbiota data generated from 16S rRNA amplicon sequencing and are log10(counts + 1) while transcripts data were generated from RNA‐seq and are counts per million (CPM). Displayed Spearman's correlations, false discovery rate < 0.05, and have <20% of zeros in 16S data. ASV labels (*x*‐axis) are family classification and amplicon sequence variant identification. All correlations are provided in Table S1

Surprisingly, only two colonic transcripts were found to be significantly altered at FDR < 0.05 across all comparisons (Table 4). Log fold difference of colonic collagen type I (and III) alpha 1 chain (*Col1a1* and *Col3a1*) were lower in D3M rats relative to ND. At a liberal FDR < 0.2, 22 additional colon transcripts were identified as differentially expressed between D3M and ND rats (Table 5). These transcripts were correlated to ASVs from the colon contents and 21 pairwise correlations were identified at FDR < 0.05 (Table S1). We further removed correlations with a high abundance of zeros, which could artificially leverage the direction and strength of the relationship. Only seven correlations remained and mostly consisted of ASVs classified to the Muribaculaceae and Oscillospiraceae families (Figure 5). Igfbp3 and Pzp transcripts positively correlated to Oscillospiraceae ASVs; Fut2, Pzp, and Gata2 positively correlated to Muribaculaceae ASVs and Susd4 negatively correlated to an ASV also classified to the Muribaculaceae family. No colonic transcripts were identified at FDR < 0.2 when comparing ND to D1M rats or D1M to D3M.

**TABLE 4:** Colon RNA transcriptional differences in male UC Davis Type 2 Diabetes Mellitus (UCD‐T2DM) rats compared to nondiabetic rats^1^

Symbol	Entrez	Log fold change	Log CPM	*F*	*p*	FDR
*Col1a1*	29393	−0.997	7.689	48.912	<0.01	0.033
*Col3a1*	84032	−0.955	8.820	43.165	<0.01	0.035

Abbreviations: *Col1a1*, colonic collagen type I alpha 1 chain; *Col3a1*, colonic collagen type III alpha 1 chain; CPM, counts per million.

^1^ Comparison between UCD‐T2DM nondiabetic rats (ND, *n* = 6) and UCD‐T2DM rats 3‐months post‐onset of diabetes (D3M, *n* = 6). Analysis conducted using the edgeR pipeline. Log fold change represents the ratio of D3M to ND groups.

**TABLE 5:** Colon RNA transcriptional differences in male UC Davis Type 2 Diabetes Mellitus (UCD‐T2DM) rats 3 months post diabetes compared to nondiabetic rats^1^

Symbol	Entrez	Log fold change	Log CPM	*F*	FDR	Description
*Adamts12*	294809	−1.32	1.177	22.526	0.139	ADAM metallopeptidase with thrombospondin type 1 motif, 12 [Source: RGD Symbol; Acc:1307323]
*Igfbp3*	24484	−1.19	6.711	25.951	0.139	Insulin‐like growth factor binding protein 3 [Source: RGD Symbol; Acc:2874]
*Gata2*	25159	−1.06	2.582	22.361	0.139	GATA binding protein 2 [Source:RGD Symbol; Acc:2664]
*Pzp*	252922	−1.02	5.586	26.349	0.139	pregnancy‐zone protein [Source:RGD SYMBOL; Acc:628643]
*Col1a1*	29393	−1.00	7.688	48.912	0.033	Collagen type I alpha 1 chain [Source: RGD Symbol; Acc:61817]
*Col6a4*	315981	−1.00	4.827	29.866	0.139	collagen, type VI, alpha 4 [Source:RGD Symbol; Acc:1564060]
*Col3a1*	84032	−0.96	8.820	43.165	0.035	Collagen type III alpha 1 chain [Source: RGD Symbol; Acc:71029]
*Aif1l*	362107	−0.95	2.570	23.604	0.139	Allograft inflammatory factor 1‐like [Source: RGD Symbol; Acc:1305081]
*Igsf10*	310448	−0.92	3.329	21.061	0.158	Immunoglobulin superfamily, member 10 [Source: RGD Symbol; Acc:735030]
*C1qtnf2*	497886	−0.90	3.452	22.427	0.139	C1q and tumor necrosis factor‐related protein 2 [Source: RGD Symbol; Acc:1561041]
*Sparc*	24791	−0.81	8.298	19.955	0.195	Secreted protein acidic and cysteine rich [Source: RGD Symbol; Acc:3742]
*Cxcl12*	24772	−0.74	4.566	23.507	0.139	C‐X‐C motif chemokine ligand 12 [Source: RGD Symbol; Acc:3651]
*Stmn1*	29332	−0.50	5.852	20.940	0.158	Stathmin 1 [Source: RGD Symbol; Acc:2992]
*Fgfr4*	25114	−0.47	5.103	23.257	0.139	fibroblast growth factor receptor 4 [Source: RGD Symbol; Acc:2612]
*Fut2*	58924	0.367	8.273	22.921	0.139	Fucosyltransferase 2 [Source: RGD Symbol; Acc:2639]
*Med21*	312849	0.402	5.584	24.831	0.139	Mediator complex subunit 21 [Source: RGD Symbol; Acc:1309836]
*Aldh2*	29539	0.463	9.486	24.040	0.139	Aldehyde dehydrogenase 2 family (mitochondrial) [Source: RGD Symbol; Acc:69219]
*Kcne3*	63883	0.495	7.682	26.004	0.139	Potassium voltage‐gated channel subfamily E regulatory subunit 3 [Source: RGD Symbol; Acc:621384]
*Abcc5*	116721	0.575	6.186	22.060	0.141	ATP binding cassette subfamily C member 5 [Source: RGD Symbol; Acc:70913]
*Gcg*	24952	0.706	7.336	25.171	0.139	Glucagon [Source: RGD Symbol; Acc:2668]
*Eva1c*	360695	0.781	4.148	27.933	0.139	Eva‐1 homolog C [Source: RGD Symbol; Acc:1307569]
*Pde9a*	191569	0.808	7.642	24.820	0.139	Phosphodiesterase 9A [Source: RGD Symbol; Acc:621035]
*Susd4*	289335	0.829	2.220	21.239	0.158	Sushi domain containing 4 [Source: RGD Symbol; Acc:1564043]
*Slc6a9*	116509	0.899	4.407	23.544	0.139	Solute carrier family 6 member 9 [Source: RGD Symbol; Acc:621243]

Abbreviation: CPM, counts per million.

^1^ Comparison between UCD‐T2DM nondiabetic rats (ND, *n* = 6) and UCD‐T2DM rats 3‐months post‐onset of diabetes (D3M, *n* = 6). Analysis conducted using the edgeR pipeline. Log fold change represents the ratio of D3M to ND groups.

Contrary to observations in the colon, several ileal transcripts were differentially regulated with the advancement of diabetes (Figure 4D). These observations were all determined at FDR < 0.05, suggesting that the ileum was considerably more affected by diabetes relative to the colon in UCD‐T2DM rats. When comparing ND versus D1M, two transcripts were differentially expressed: *Asz1* was greater and *Slc30a10* was lower in D1M rats relative to ND rats (Figure 4D). When comparing ND versus D3M, 1683 transcripts were differentially expressed (Figure 4D). There were 354 transcripts that were differentially expressed between D1M and D3M rats (Figure 4D), but 319 of these overlapped with comparisons between ND rats (Table S2). Pathway analysis of the overlapping transcripts showed a significant enrichment of pathways related to protein and carbohydrate digestion and absorption (FDR < 0.1; Table 6). As ND animals were significantly younger than their diabetic counterparts, we also analyzed the age‐matched D1M versus D3M comparison. Therefore, the remaining 33 transcripts were considered the strongest indicators of differential transcript expression associated with diabetes progression in the ileum (Figure 4E). Of these, *Sptlc3*, *Enpp7*, *Slc7a15*, and *Kctd14* had greater than twofold changes. These 33 transcripts did not correlate to any ileum ASVs at FDR < 0.05 (Table S3) and resulted in no significant pathway enrichment, even at FDR < 0.2.

**TABLE 6:** Pathway analysis of transcripts differing between D3M rats and both ND and D1M rats^1^

Category	Term	Count %	Symbols	List total	Pop hits	Pop total	Fold enrichment	FDR
rno04974: Protein digestion and absorption	9	2.8	Slc6a19, Slc3a2, Atp1a1, Dpp4, Atp1b1, Slc7a7, Slc15a1, Ace2, Xpnpep2	137	89	7749	5.72	0.0293
rno04973: Carbohydrate digestion and absorption	6	1.9	Slc2a2, Atp1a1, Slc2a5, Slc37a4, Atp1b1, Slc5a1	137	41	7749	8.28	0.0652
rno04144: Endocytosis	13	4.1	Igf2r, Snx32, Ehd1, Chmp4c, Washc2c, Prkcz, Mdm2, Asap1, Arfgap3, Dnm2, Smap1, Arpc2, Epn2	137	270	7749	2.72	0.1695
rno04978: Mineral absorption	5	1.6	Slc6a19, Trpm6, Atp1a1, Atp1b1, Slc5a1	137	39	7749	7.25	0.2169
rno04976: Bile secretion	6	1.9	Atp1a1, Abcg2, Slc51b, Slc51a, Atp1b1, Slc5a1	137	71	7749	4.78	0.2983
rno04931: Insulin resistance	7	2.2	Slc2a2, Slc27a4, Trib3, Prkag2, Ptprf, Prkcz, Irs2	137	110	7749	3.60	0.3940
rno01100: Metabolic pathways	32	10	Fut1, Anpep, Dgat2, Ampd3, Papss2, Acsl5, B3galnt1, Polr3c, Dhrs9, Lpin3, Ahcyl2, Etnk1, Aldob, Khk, Dlst, Rdh16, Acat1, Bckdhb, Plcd1, Chpf, Pmm2, Fahd1, St6galnac1, Cyp3a9, Coq5, Gpat3, Chdh, Btd, B4galt6, Hsd17b4, Hsd17b2, Pla2g12b	137	1285	7749	1.41	0.8438

Abbreviation: FDR, false discovery rate.

^1^ UC Davis Type 2 Diabetes Mellitus rats were non‐diabetic (ND, *n* = 6), had recent diabetes (2‐week post‐onset of diabetes; D1M, *n* = 6), or 3‐month post‐onset of diabetes (D3M, *n* = 6). Transcript expression determined by RNAseq analysis. Differential abundance between rat groups was assessed using edgeR pipeline. Pathway analysis conducted by the DAVID Bioinformatics Resource 6.8. Only pathways with *p* < 0.05 are provided.

## 4 DISCUSSION

This is the first report describing diabetes‐associated alterations in morphology and transcriptional abundances of the ileum and colon in the UCD‐T2DM rat model. We found a linear increase of the ileal villi length and crypt depth, and colonic crypt depth that followed the advancement of diabetes in this rat model of diabetes. This finding is in agreement with other studies examining various rodent models of diabetes. For example, Adachi et al. reported an increase of villi length from small intestine sections in both Goto–Kakizaki (GK) and Otsuka Long‐Evans Tokushima Fatty rats compared with their respective nondiabetic control; in addition to increased small intestine villi length in streptozotocin‐induced (STZ) diabetes Wistar rats relative to control Wistar rats (Adachi et al., 2003). While the small intestine region was not defined in Adachi et al., Isah and Masola (2017) found no difference in ileal villi height in STZ‐treated Sprague Dawley rats compared controls; however, increases in duodenal and jejunal villi heights were observed in the diabetic rats. STZ doses and duration of diabetes differed in these studies, which may explain the conflicting observations. Nonetheless, this line of evidence suggests that proximal regions of the small intestine are subject to villi expansion in diabetic animals. Differences associated with diabetes in the ileum are less clear. Increases in ileal villi height have been reported in STZ‐induced diabetic rats compared with controls (Isah & Masola, 2017; Thulesen et al., 1999), however, in another study there were no reported differences or reduced ileal villi heights in STZ‐treated animals (Xue et al., 2019). Limited data have been collected in mono‐ and polygenic rodent models of diabetes. In one study, Pereira et al. recently observed longer ileal villi and decreased ileal crypt depth in 4‐month old male GK rats compared to male Wistar rats (Pereira et al., 2021). On balance, data from our study points to an overall expansion of the gut mucosal layer during the advancement of untreated diabetes; however, the mechanisms underlying this expansion are not known and the consequence of these morphological changes will need further investigation.

Untreated diabetes in the UCD‐T2DM rat model leads to progressive loss of energy stores and skeletal muscle mass, as indicated by the decrease in total body weight in rats with 3 months post‐onset of diabetes. Therefore, one possibility is that the expansion of the villi and crypt depth may be a response to a perceived nutrient deficiency and an attempt by the organism to extract more energy or nutrients from the gastrointestinal tract. Contrary to this hypothesis, intestinal atrophy has been observed in humans with depleted nutritional status and in animals undergoing food deprivation and/or restriction (Genton et al., 2015). While there are obvious differences between glucose deprivation versus food restriction (e.g., hyperglycemia, gut hormonal response to feeding, etc.), uncontrolled diabetes still mimics a state of catabolism since circulating glucose is less able to be utilized by peripheral tissues and is subsequently excreted in urine. Compared with age‐matched nondiabetic Sprague Dawley rats, UCD‐T2DM rats consume a greater amount of chow and then linearly increase their energy intake 100 days post diabetes (Cummings et al., 2008). Thus, the proliferative effect on both the ileum and colon mucosal layer may primarily be due to the increased ingestion and passage of nutrients through the gastrointestinal tract rather than an effect of diabetes per se. A possible mechanism for this expansion may be via nutrient‐stimulated secretion of glucagon‐like peptide‐2, which is known to have a proliferative effect on the intestinal epithelia (Drucker et al., 1996; Drucker & Yusta, 2014).

We observed compositional differences in the colonic microbiota population concurrent with the advancement of diabetes; however, we could not conclusively identify whether diabetes impacts the ileum microbiota. The inconclusive results in the ileum were due to low sample depth that rendered half of the D3M samples unusable for statistical analysis. As age is a known modifier of the gut microbiota (Sovran et al., 2019; Wu et al., 2021), we also cannot conclusively distinguish whether the differences between the younger ND rats and the older diabetic rats are due to diabetes or age. In a previous study, compositional differences in between UCD‐T2DM rats with an early or late diabetes signature were primarily driven by an increase in species within the Bacteroidetes phyla (Piccolo et al., 2018). While we found compositional differences in the current study, the change in Bacteroidetes taxa was not found. This could be due to the fact that the previous study examined cecal contents compared to ileum and colon contents in the study herein. Furthermore, the prior study utilized shotgun metagenomics sequencing, which requires much deeper sequencing relative to 16S rRNA amplicon sequencing. Regardless, we interpret these data to confirm the effect of diabetes on the broader bacterial composition (i.e., beta‐diversity) in this model, but confirmation of altered taxonomy will require additional studies with much deeper sequencing. Still, we found a decrease in ASVs classified to the *Akkermansia* genus in colon contents from D3M rats comparing to D1M rats, which concurs with previous reports suggesting an association between *Akkermansia* and host metabolic health. Greater *Akkermansia muciniphila* abundances have been associated with reduced adiposity (Everard et al., 2013), improved host glucose homeostasis (Yoon et al., 2021), and intestinal barrier integrity (Justus et al., 2015). Other studies have indicated that supplementation with intact or inactivated *A. muciniphila* improves host metabolism (Everard et al., 2013; Plovier et al., 2017), which may be an intriguing therapeutic option for this model of diabetes.

Diabetes and/or hyperglycemia has been shown to increase gut permeability and inflammation in several rodent models of diabetes compared to nondiabetic controls (Ahmad et al., 2017; Cani et al., 2008). Much of these effects have been associated with dysbiosis of the gut microbiota, leading to the unintended absorption of small microbial molecules into circulation (e.g., lipopolysaccharide), which stimulate immune and inflammatory responses at various tissues. More recent evidence has suggested that hyperglycemia is the driver behind this mechanism (Thaiss et al., 2018), therefore, we assessed whether colonic gene expression of claudins and several inflammatory markers would be altered by the progression of diabetes in UCD‐T2DM rats. Contrary to our hypothesis, we found no evidence of changes in gene expression in these parameters with the advancement of diabetes. While protein levels of claudins and other tight junction‐related proteins have been shown to be altered with obesity and insulin resistance (Ahmad et al., 2017), the exact regulatory mechanism behind this relationship has not been conclusively established. Epithelial metabolism may play an important role in regulating barrier function as more recent evidence has suggested that a switch from aerobic respiration of short‐chain fatty acids to anaerobic oxidation of glucose may directly regulate barrier function via changes in cellular oxygen concentrations (Kelly et al., 2015). As gene expression is likely an insensitive measure of barrier function, current work is underway in this model of type 2 diabetes to directly measure permeability and potential regulatory mechanisms.

After finding null results in the targeted gene expression analysis, we employed RNAseq to survey both the ileum and colon for transcriptional changes associated with the advancement of diabetes. Surprisingly, the upper colon continued to show very little genetic changes between an early versus late duration of diabetes (D1M vs. D3M) as no transcripts remained significant after adjusting for multiple comparisons. While these results need to be confirmed in additional studies, the upper bowel appears to be more susceptible than the colon to the effects of advancing duration of diabetes in the UCD‐T2DM rat model. Still, two transcripts related to collagen synthesis (*Col1a1* and *Col3a1*) were reduced in D3M rats compared to ND rats, suggesting that the advancement of diabetes weakens the structural strength and integrity of the colon. In contrast to our results, Kandemir et al. reported a thickening of the subepithelial collagen layer in rectal biopsies from diabetic adults compared to nondiabetic adults (Kandemir et al., 1995) while other reports from STZ‐treated rats indicate both thickening and stiffening of the colonic wall (Zhao et al., 2003). The UCD‐T2DM rat has a polygenic origin of diabetes and may be susceptible to fibrosis early in its lifespan; thus, the decrease *Col1a1* and *Col3a1* in an advanced state of diabetes could be a compensation mechanism for excess colonic fibrosis in this model. We did not directly measure fibrosis in this study, so this hypothesis will need to be explored further in additional studies.

In ileal tissue, *Enpp7* (ectonucleotide pyrophosphatase/phosphodiesterase family member 7; also known as alkaline sphingomyelin phosphodiesterase) and *Sptlc3* (serine palmitoyltransferase, long chain base subunit 3) were the two transcripts that had the largest differences between D1M and D3M rats. Interestingly, these transcripts are both involved in intestinal ceramide and sphingomyelin homeostasis. Enpp7 digests luminal sphingomyelin and its activity has been associated with diseases affecting the colon, for example, colitis and colorectal cancer (Hertervig et al., 1997; Sjöqvist et al., 2002). Enpp7 has also been shown to have antiproliferative properties (Hertervig et al., 2003) and could be downregulated in the UCD‐T2DM rat to facilitate villi and crypt depth expansion in this model. Serine palmitoyltransferase, on the other hand, is the first step in cellular ceramide synthesis and it also required for barrier function and integrity. Recently, knockdown of the intestinal SPT complex via an intestinal‐specific Sptlc2 deletion resulted in a bacterial infiltration of the mucosa, reduced microvilli height, and reduction of levels of E‐cadherin, suggesting that SPT has a critical role in barrier function (Li et al., 2018). Overall, Sptlc3 and Enpp7 had the largest transcriptional changes and seem to support a proliferative effect of the mucosa during the advancement of diabetes.

This is the first paper to report diabetes‐associated changes in the gastrointestinal tract of UCD‐T2DM rats, a polygenic rodent model of spontaneous and progressive T2DM. That said, a limitation is that the UCD‐T2DM rat does not have a metabolically healthy control with a similar genetic background since prediabetic UCD‐T2DM rats are obese and insulin resistant. Lean Sprague Dawley rats have been previously used as a metabolic healthy and lean comparison, but we have noted clear differences in the composition of the cecal microbiota between lean Sprague Dawleys and UCD‐T2DM rats regardless of their diabetes status, indicative of a strain effect (Piccolo et al., 2018). As genetic background is known to significantly impact the composition of the gut microbiota (Carmody et al., 2015), we chose to study younger UCD‐T2DM rats that had normal fasting blood glucose concentrations and lower fat mass than recently diabetic animals. This, of course, made age a potentially confounding factor and comparisons between the ND and older diabetic groups should be taken with caution. In addition, we did not observe any diabetes‐specific differences in colon tight junction expression as measured by rtPCR and no major alteration of the colon transcriptome with the advancement of diabetes. We examined the proximal colon, just distal to the cecum, due to the fact that we previously observed clear differences in the cecal microbiota from this model (Piccolo et al., 2018). Future studies may need to differentiate between proximal and distal colon observations as some evidence suggests that fermentation of SCFA differs between the proximal and distal regions (Topping & Clifton, 2001).

In conclusion, advancement of untreated diabetes in the UCD‐T2DM rat results in an increase in the mucosal villi and crypt depth in the ileum, and increased crypt depths in the colon, which we speculate derives from signals associated with host metabolic status and differences in food intake (i.e., diabetic hyperphagia) in this model as diabetes progresses. The advancement of diabetes resulted in very minor transcriptional changes in the colon, while numerous transcriptional differences were identified in the ileum, suggesting that the upper bowel may be more susceptible to the effects of diabetes. *Sptlc3* and *Enpp7* exhibited the largest transcriptional changes associated with the progression of untreated diabetes suggesting that changes in ceramide/sphingolipid metabolism to support the enlargement of the mucosal luminal interface. While requiring future studies that fully control for food intake and age, the current results strongly support emerging evidence that host metabolic health and diabetes status have diet‐independent effects on intestinal growth and function, and can also influence regional gut microbiota populations.

## CONFLICT OF INTEREST

S.H. Adams is the founder and principal of XenoMed, LLC, which is focused on research and discovery unrelated to the studies herein. All other authors declare no conflict of interest, financial, or otherwise.

## AUTHOR CONTRIBUTIONS

Brian D. Piccolo, Peter J. Havel, and Sean H. Adams conceived and designed research; Brian D. Piccolo, James L. Graham, Ping Kang, Christopher E. Randolph, Laxmi Yeruva, Renee Fox, Tanya LeRoith, and Kimber L. Stanhope performed experiments; Brian D. Piccolo, Kimber L. Stanhope, Michael S. Robeson, and Becky Moody analyzed data; Brian D. Piccolo interpreted results of experiments, prepared figures, and drafted manuscript; all authors approved final version of the manuscript.

## Supporting information



Table S1

Table S2

Table S3
